# Association Between Kinetics of Early Biofilm Formation and Clonal Lineage in *Escherichia coli*

**DOI:** 10.3389/fmicb.2019.01183

**Published:** 2019-05-31

**Authors:** Saskia-Camille Flament-Simon, Marion Duprilot, Noémie Mayer, Vanesa García, María Pilar Alonso, Jorge Blanco, Marie-Hélène Nicolas-Chanoine

**Affiliations:** ^1^Laboratorio de Referencia de Escherichia coli, Departamento de Microbioloxía e Parasitoloxía, Facultade de Veterinaria, Universidade de Santiago de Compostela, Lugo, Spain; ^2^Service de Microbiologie, Hôpital Beaujon, AP-HP, Clichy, France; ^3^IAME, UMR 1137, INSERM, Université Paris Diderot, Paris, France; ^4^Unidade de Microbioloxía, Hospital Universitario Lucus Augusti, Lugo, Spain

**Keywords:** *E. coli*, early biofilm formation, phenotypes, virulence factors, clones, ST131, ST127, ST141

## Abstract

**Background:**

*Escherichia coli* biofilm formation has mostly been assessed in specific pathogenic *E. coli* groups. Here, we assessed the early biofilm formation (EBF), i.e., adhesion stage, using the BioFilm Ring Test^®^ on 394 *E. coli* clinical isolates (EC) [196 consecutively isolated (CEC) in 2016 and 198 ESBL-producing *E. coli* (ESBLEC) isolated in 2015]. Then, biofilm-forming ability was contrasted with phylogroups, clonotypes (*fumC*-*fimH*), and sequence types (STs), all being used to define clones, virulence factors (VF), and FimB.

**Result:**

According to both biofilm production levels at 2, 3, and 5 h, and EBF kinetics over 5 h, CEC and ESBLEC isolates segregated into three EBF groups: strong (G1), moderate (G2), and weak (G3) producers. At 2 h, strong producers were more frequent among CEC (*n* = 28; 14.3%) than among ESBLEC (*n* = 8; 4%) (*P* = 0.0004). As CEC and ESBLEC isolates showed similar individual EBF kinetics in each group, a comparison of isolate features between each group was applied to gathered CEC and ESBLEC isolates after 2 h of incubation, 2 h being the most representative time point of the CEC and ESBLEC isolate segregation into the three groups. Phylogroup B2 displayed by 51.3% of the 394 isolates was more frequent in G1 (77.8%) than in G3 (47.6%) (*P* = 0.0006). The 394 isolates displayed 153 clones, of which 31 included at least three isolates. B2-CH14-2-ST127, B2-CH40-22-ST131, B2-CH52-5/14-ST141, and E-CH100-96-ST362 clones were associated with G1 (*P* < 0.03) and accounted for 41.7% of G1 isolates. B2-CH40-30-ST131 clone was associated with G3 (*P* < 0.0001) and accounted for 25.5% of G3 isolates. VF mean was higher among G1 than among G3 isolates (*P* < 0.001). FimB-P2 variant was associated with G1 (*P* = 0.0011) and FimB-P1 variant was associated with G3 (*P* = 0.0023). Clone, some VF, and FimB were associated with EBF, with clonal lineage being able to explain 72% of the variability of EBF.

**Conclusion:**

Among our 394 isolates, <10% are able to quickly and persistently produce high biofilm levels over 5 h. These isolates belong to a few clones previously described in various studies as dominant gut colonizers in mammalians and birds and comprised the B2-CH40-22-ST131 clone, i.e., the ancestor of the globally disseminated B2-CH40-30-ST131 clone that is the dominant clone among the weak biofilm producers.

## Introduction

Since the first definition of biofilms provided by Costerton et al. (1978) 30 years ago, it is well established that the majority of bacteria found in nature exists attached to surfaces within the structured biofilm ecosystem (Costerton et al., 1978; Hall-Stoodley et al., 2004). Bacterial biofilms are known for their resistance to antibiotics, disinfectants, and components of the innate and adaptative inflammatory defense system of the body (Høiby et al., 2011). Accordingly, biofilm-growing bacteria cause chronic infections, persisting inflammation, tissue damage, and foreign body infections (Høiby et al., 2011). Thus, it was shown that persistence of staphylococcal infections related to foreign bodies is due to biofilm formation (Sabaté Brescó et al., 2017). Likewise, chronic *Pseudomonas aeruginosa* lung infections in cystic fibrosis patients are caused by biofilm-growing mucoid isolates (Høiby et al., 2010).

Concerning *Escherichia coli*, which can exist as a harmless commensal in the mammalian digestive tract and as a pathogen causing significant morbidity and mortality worldwide, its ability to form biofilm has been extensively studied from non-pathogenic *E. coli* K12 strains (Beloin et al., 2008). With regard to pathogenic *E. coli*, biofilm involvement in pathogenesis has been well defined in diarrheagenic *E. coli*, notably enteroaggregative *E. coli* (Sheikh et al., 2001; Sherlock et al., 2004; Schiebel et al., 2017) and in adherent-invasive *E. coli* that have been implicated in the origin and perpetuation of Crohn’s disease (Martinez-Medina et al., 2009b). Production of biofilm by extraintestinal pathogenic *E. coli* (ExPEC) was mostly assessed in uropathogenic *E. coli* (Watts et al., 2010; Ponnusamy et al., 2012; Agarwal et al., 2013; Tapiainen et al., 2014). All these studies highlighted various biofilm formation phenotypes among uropathogenic *E. coli* including isolates obtained from patients with an indwelling catheter. Assessment of biofilm formation focusing on the pandemic extended-spectrum β-lactamase (ESBL)-producing and multidrug-resistant ST131 *E. coli* clone (Nicolas-Chanoine et al., 2014) showed highly contrasting results: very low levels (Novais et al., 2012) and moderate levels (Hussain et al., 2014) of biofilm formation. Sarkar et al. (2016) studying ST131 isolates expressing or non-expressing type 1 fimbriae showed that biofilm growth depended on type 1 fimbriae expression and assay conditions. We recently showed different phenotypes among ST131 isolates according to their *fimH* allele encoding adhesins of type 1 fimbriae, namely, significant higher levels of early biofilm production by isolates of ST131 *H*22 subclone than those of ST131 *H*30 subclone (Nicolas-Chanoine et al., 2017).

In the present study, we assessed biofilm formation in 394 clinical isolates obtained from different sources in two geographically distant hospitals and during the same time periods. We focused on the early biofilm formation (EBF), i.e., at adhesion stage, because this stage is the key stage in the colonization process of abiotic and biotic surfaces, body biotic surfaces being the epithelia of the various organs for the ExPEC isolates and mucus layers in gut, i.e., the *E. coli* natural habitat, for all *E. coli* populations (Ellermann and Sartot, 2018). For this, we used the BioFilm Ring Test^®^, a microbead immobilization assay adapted from Chavant et al.’s (2007) method that was shown to have a good concordance with the crystal violet method (Di Domenico et al., 2016) and offers a reproducible and quantifiable measure of the first step of biofilm formation. Expecting, as previously described, different biofilm formation phenotypes among our isolates, the main goal of this study was to molecularly characterize the isolates that enabled us to show that some traits are associated with the different phenotypes.

## Materials and Methods

### Bacteria

Two collections of *E. coli* clinical isolates were studied. The first collection consisted of 196 non-duplicate (one isolate per patient) *E. coli* consecutively isolated (CEC) in 2016 from two hospitals (Lugo hospital in Spain, *n* = 100, and Beaujon hospital in France, *n* = 96). This collection that comprised 13 ESBL-producing isolates came from different sources: 146 from urine, 22 from blood, 5 from bile, 3 from ascitic fluid, 6 from abscesses, and 14 from various other sources. As ESBL production is currently one of the most important worldwide threatening mechanisms of antibiotic resistance in *E. coli* clinical isolates, we studied a second collection consisting of 198 non-duplicate ESBL-producing *E. coli* (ESBLEC) isolates obtained from the same two hospitals in 2015 (Lugo hospital, *n* = 99, and Beaujon hospital, *n* = 99). This collection comprised 147 isolates from urine, 26 from blood, 7 from bile, 4 from ascitic fluid, 2 from abscesses, and 12 from various other sources. ESBL production detected by the double disk synergy test (Jarlier et al., 1988) performed in the two hospitals had been confirmed by specific ESBL PCR and sequencing as previously described (Leflon-Guibout et al., 2008; Mora et al., 2018).

### Determination of Early Biofilm Formation

The kinetics of EBF was assessed using the BioFilm Ring Test^®^ (BioFilm Control, Saint-Beauzire, France) according to the manufacturer’s recommendations. Briefly, each isolate was twice sub-cultured on brain heart infusion (BHI) agar (Benton Dickinson, Le Pont-de-Claix, France) at 37°C for 24 h. Three colonies of the second subculture on BHI agar were suspended in BHI broth (Biofilm Control). Suspension was standardized to an optical density at 600 nm of 1.00 ± 0.05 (Ultrospec10: Biochrom, Cambridge, United Kingdom) and then diluted at 1:250 in BHI broth to obtain a final concentration of approximately 10^6^ UFC/ml. The bacterial suspension was supplemented (1% vol/vol) with magnetic microbeads (TONER 4, Biofilm Control) and 200 μl of the mix was deposited in two wells of three polystyrene 96-well microtiter plates that were incubated for 2, 3, and 5 h, respectively. At the end of each incubation time, 100 μl of liquid contrast solution (LIC001, Biofilm Control) was added on the top of each well and the microplate was put on a magnetic block for 1 min. After magnet contact, free beads were attracted toward the center of each well, forming a brown spot, while beads embedded in biofilms were blocked and remained undetectable. Each microplate was scanned using a BioFilm Control plate reader. The intensity of the spot was analyzed using the BioFilm Ring Test^®^-software version 3.0.3 and expressed as a biofilm formation index (BFI) with values ranging from 20 (non-formation of biofilm) to 0 (high formation of biofilm) that is inversely proportional to attached bacteria that block the beads. BFI values ≤ 5 mean that the isolates are strong biofilm producers; BFI values between 6 and 14, moderate producers; BFI values between 15 and 19, weak producers; non-producers display a BFI value of 20. Each isolate was tested in three independent experiments and the average of the six measures was used as the final BFI value. For each experiment, isolates S250 and 39 previously described with the method carried out in the present study as strong and negative producers of biofilm, respectively, were used as controls (Nicolas-Chanoine et al., 2017). We also included BHI broth without bacteria as negative control.

### Molecular Characterization of Isolates

As previously described, phylogroups (Clermont et al., 2013), sequence types (STs) according to the MLST scheme of Achtman^1^, *fumC* (C), and *fimH* (H) clonotypes (Weissman et al., 2012), and virulence factors (VF) (Mora et al., 2018) were determined in all 394 *E. coli* isolates. Clones were characterized by using the association of phylogroup, clonotype, and ST. Among the 394 isolates, 127 (approximately one-third of the 394 isolates) were randomly selected by using the RAND function (Microsoft Excel) system for the *fimB* gene analysis. The *fimB* gene was amplified with specific primers (FimB F: 5′-AGCATGGCGTTTGTATGG-3′; FimB R: 5′-CCCTGGTATCTCAACTATCTCT-3′) and sequenced as previously described (Nicolas-Chanoine et al., 2017). When the *fimB* gene was disrupted, the detection of the previously described *IS*3-like in *H*30 R isolates (Totsika et al., 2011; Nicolas-Chanoine et al., 2017) was performed by PCR using specific primers designed in this study (FimBw F: 5′-AGCATGGCGTTTGTATGG-3′; IS R: 5′-CTGAATGTGATGTGCCGATG-3′). Phylogenetic tree of FimB variants was constructed by the UPGMA method of MEGA 6.

### Statistical Analysis

Analysis of variance (ANOVA) test was performed to compare the mean number of virulence genes and the association of variables with biofilm formation phenotypes. Dichotomous variables were described using enumeration and percentage, and compared using two-tailed Fisher’s exact test. *P*-values < 0.05 were considered statistically significant. Correlation between quantitative variables was assessed by Pearson correlation coefficient, and the assessment of repeatability was via the Bland and Altman (1986) statistical method. All analyses were carried out by XLSTAT statistical software^2^.

### Nucleotide Sequence Accession Numbers

The 34 *fimB* gene sequences have been registered in GenBank database under the following accession numbers: MK301552 to MK301585.

## Results

### Kinetics of Early Biofilm Formation

To assess the repeatability of the duplicate determination of biofilm formation performed on each of the 394 isolates at each time, we calculated Pearson’s correlation coefficient and Bland and Altman (1986) analysis between the BFI obtained for each determination. Pearson’s correlation coefficient was high and constant over time: 0.96 at 2 h, 0.96 at 3 h, and 0.95 at 5 h (Supplementary Figure S1A), and the Bland and Altman (1986) analysis showed that only 3.4% of values at 2 h, 5.5% at 3 h, and 6.8% at 5 h exceed the established standard deviation (Supplementary Figure S1B).

Biofilm formation index values obtained at each time point (2, 3, and 5 h) showed a distribution of both CEC and ESBLEC isolates in the three levels of biofilm production (strong, moderate, and weak production) defined according to the BFI value ranges. After 2 h of incubation, strong producers (BFI: 0–5) and moderate producers (BFI: 6–14) were more frequent among CEC isolates than ESBLEC isolates (*P* = 0.004 and *P* = 0.0189, respectively), whereas weak and non-producers (BFI: 15–20) were more frequent among ESBLEC isolates than among CEC isolates (*P* < 0.0001) (Table 1). After 3 h of incubation, such significant differences were still observed between CEC and ESBLEC isolates for the strong and weak producers but not for the moderate producers. After 5 h of incubation, no significant difference was observed between CEC and ESBLEC for the three types of producers.

**Table 1:** Distribution of CEC and ESLEC isolates according to the biofilm index (BFI) values after 2, 3, and 5 h of incubation.

BFI range	Number (%) of isolates		*P*-value^a^	Number (%) of isolates		*P*-value^a^	Number (%) of isolates		*P*-value^a^
	At 2 h			At 3 h			At 5 h		
	CEC *n* = 196	ESBLEC *n* = 198		CEC *n* = 196	ESBLEC *n* = 198		CEC *n* = 196	ESBLEC *n* = 198	
0–5	28 (14.3)	8 (4.0)	0.0004	36 (18.4)	11 (5.6)	< 0.0001	71 (36.2)	70 (35.4)	0.9164
6–14	20 (10.2)	8 (4.0)	0.0189	39 (19.9)	27 (13.6)	0.1064	41 (20.9)	45 (22.7)	0.7150
15–20	148 (75.5)	182 (91.9)	< 0.0001	121 (61.7)	160 (80.8)	< 0.0001	84 (42.9)	83 (41.9)	0.9188

*^*a*^Two-tailed *P*-values by Fisher’s exact test.*

**FIGURE 1:**
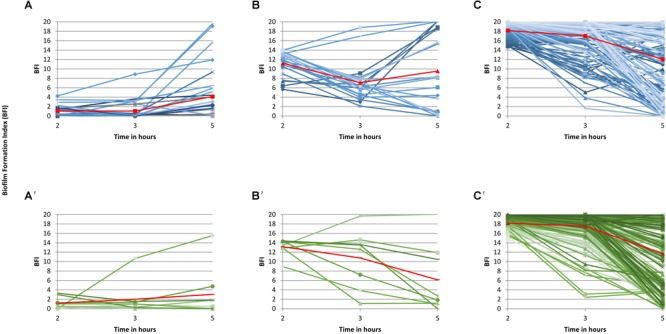
Individual biofilm formation trajectories over time for the 394 isolates. Individual biofilm formation index (BFI) and average BFI values at each time point (2, 3, and 5 h) are represented as blue lines and a red connected line, respectively, for CEC isolates **(A–C)** and as green lines and a red connected line, respectively, for ESBLEC isolates **(A’–C’)**. **(A,A’)** present the biofilm kinetics of the strong biofilm producers at 2 h (BFI≤5), **(B,B’)**, the biofilm kinetics of the moderate producers at 2 h (BFI>5–≤14), and **(C,C’)**, the biofilm kinetics of the weak (BFI>14 and <20) and the non-producers (BFI = 20) at 2 h.

The study of individual BFI trajectories of the isolates of the three 2-h groups over time showed that both CEC and ESBLEC isolates displayed three distinct EBF kinetics. As indicated in Figure 1A,A’, most of the 28 CEC (27 non-ESBL producers) and the 8 ESBLEC strong biofilm producers at 2 h (average BFI: 1 for the two collections) remained strong producers after 3 h (average BFI: 1 and 2, respectively) and 5 h (average BFI: 4 and 3, respectively) of incubation. Accordingly, these isolates were classified into group 1 (G1) corresponding to quickly and persistently strong biofilm producers. As indicated in Figure 1B,B’, the 20 CEC and 8 ESBLEC moderate producers at 2 h, with average BFI values of 11 and 13, respectively, displayed a wider variability of BFI values at 3 and 5 h than the strong producers. However, their average BFI at 3 h (7 and 11, respectively) and at 5 h (10 and 6, respectively) led us to classify them as mostly moderate producers over the study period [group 2 (G2)]. As indicated in Figure 1C,C’, the 148 CEC and 182 ESBLEC weak producers at 2 h (average BFI: 18 for CEC and ESBLEC) also displayed a wide variability of BFI values at 3 and 5 h with a notable part of isolates becoming strong producers at 5 h. However, the average BFI values were 17 for CEC and 18 for ESBLSE isolates at 3 h, and 12 for CEC and ESBLEC isolates at 5 h. Accordingly, we classified these isolates into group G3 corresponding to weak biofilm producers. We compared the individual BFI trajectories of all CEC isolates and CEC isolates over time without the 13 ESBL-producing isolates identified in this collection and found no significant difference (data not shown).

Figure 1 shows that time point 2 h was the most representative time point of the division of our *E. coli* population into three groups. Accordingly, the comparisons between G1, G2, and G3 isolates will be made at time point 2 h in the rest of the study. Furthermore, the similarity of the individual biofilm formation kinetics of the CEC and ESBLEC isolates within each group led us to characterize altogether the CEC and ESBLEC isolates of each group in the rest of the study.

### Characterization of Phylogroups, Sequence Types, and Clones

#### Phylogroups

**FIGURE 2:**
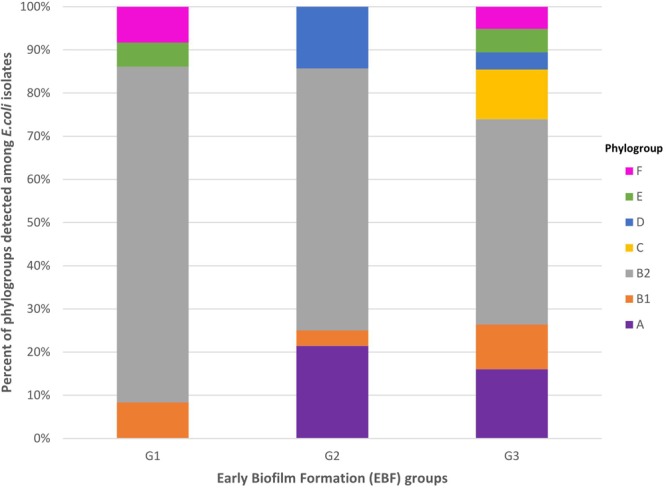
Distribution of phylogroups A, B1, B2, C, D, E, and F among G1, G2, and G3 isolates. G1: strong producers, G2: moderate producers, and G3: weak producers. Isolates belonging to phylogroup B2 were more frequent among G1 isolates (77.8%) than among G3 isolates (47.6%) (*P* = 0.0006). By contrast, isolates belonging to phylogroups A and C were significantly more frequent among G3 isolates than among G1 isolates (A: 16.1 vs. 0%, *P* = 0.0098; C: 11.5 vs. 0%, *P* = 0.0419).

Most of the 394 isolates (51.3%) were assigned to phylogroup B2. The remaining isolates were distributed into phylogroups A (15.0%), B1 (9.6%), C (9.6%), E (5.1%), F (5.1%), and D (4.3%). Isolates belonging to phylogroup B2 were predominant among G1, G2, and G3 isolates (Figure 2). The seven phylogroups (A, B1, B2, C, D, E, and F) were detected among G3 isolates, four (A, B1, B2, and D) among G2 isolates, and four (B1, B2, E, and F) among G1 isolates (Figure 2). Isolates belonging to phylogroup B2 were more frequent among G1 isolates (77.8%) than among G3 isolates (47.6%) (*P* = 0.0006) (Supplementary Table S1). By contrast, isolates belonging to phylogroups A and C were significantly more frequent among G3 isolates than among G1 isolates (A: 16.1% vs. 0%, *P* = 0.0098; C: 11.5% vs. 0%, *P* = 0.0419) (Supplementary Table S1).

#### Sequence Types

**FIGURE 3:**
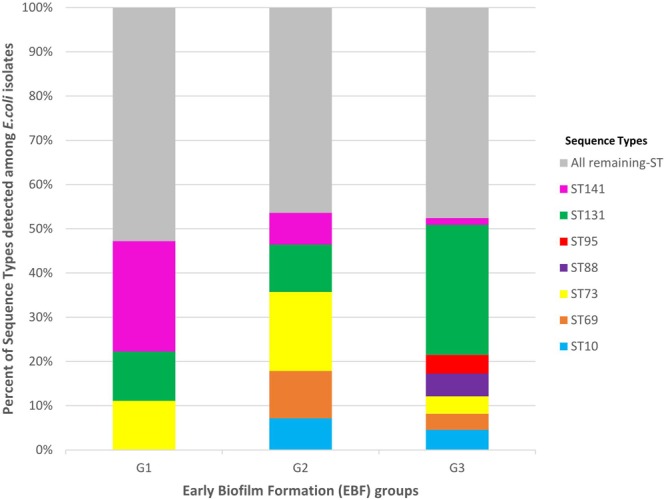
Distribution of the most frequent sequence types (STs) among G1, G2, and G3 isolates. G1: strong producers, G2: moderate producers, and G3: weak producers. ST141 was more frequent among G1 isolates than among G3 (25.0 vs. 1.5%, *P* < 0.0001). By contrast, ST131 was significantly more frequent among G3 isolates than among G1 isolates (29.4 vs. 11.1%, *P* = 0.0289).

The 394 *E. coli* isolates displayed 99 STs. The most frequent STs accounting for more than half (52.0%) of the isolates were ST131 (26.4%), ST73 (5.6%), ST10 (4.3%), ST88 (4.3%), ST141 (4.1%), ST69 (3.8%), and ST95 (3.6%). The distribution of the seven most frequent STs among G1, G2, and G3 isolates is presented in Figure 3. These seven STs were displayed by G3 isolates whereas five were displayed by G2 isolates (absence of ST88 and ST95) and only three (ST131, ST141, and ST73) were displayed by G1 isolates. Among the three STs shared by the three groups, ST131 was significantly more frequent among G3 isolates than among G1 isolates (29.4% vs. 11.1%, *P* = 0.0289). By contrast, ST141 was more frequent among G1 isolates than among G3 (25.0% vs. 1.5%, *P* < 0.0001) (Supplementary Table S2).

#### Clones

**FIGURE 4:**
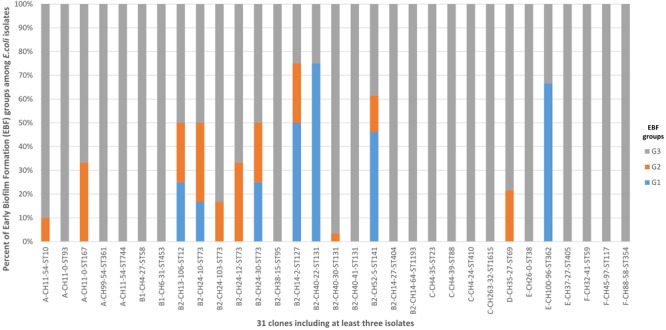
Distribution of the 31 clones including at least three isolates among G1, G2, and G3. G1: strong producers, G2: moderate producers, and G3: weak producers. Five clones (B2-CH14-2-ST127, B2-CH40-22-ST131, B2-CH52-5-ST14, B2-CH52-14-ST141, and E-CH100-96-ST362) were associated with G1 and accounted for 41.7% of G1 isolates, 10.7% of G2 isolates (*P* = 0.0105), and 2.4% of G3 isolates (*P* < 0.0001). Only the B2-CH40-30-ST131 clone was associated with G3, accounting for 25.5% of G3 isolates, 10.7% of G2 isolates (*P* = 0.1068), and 0% of G1 isolates (*P* < 0.0001).

A total of 153 clones were identified among the 394 isolates, with 31 of them including at least 3 isolates (Figure 4 and Supplementary Table S3) and 6 including at least 10 isolates: B2-CH40-30-ST131 (87 isolates), D-CH35-27-ST69 (14 isolates), B2-CH52-5-ST141 (13 isolates), C-CH4-39-ST88 (11 isolates), A-CH11-54-ST10 (10 isolates), and B2-CH40-41 ST131 (10 isolates). The 36 G1 isolates belonged to 26 clones (Supplementary Table S4). These 26 clones were also found in 25% of the G2 isolates (*P* < 0.0001) and in only 5.2% of the G3 isolates (*P* < 0.0001) (Supplementary Table S4).

Five clones (B2-CH14-2-ST127, B2-CH40-22-ST131, B2-CH52-5-ST14, B2-CH52-14-ST141, and E-CH100-96-ST362) were associated with G1 and accounted for 41.7% of G1 isolates, 10.7% of G2 isolates (*P* = 0.0105), and 2.4% of G3 isolates (*P* < 0.0001) (Supplementary Table S3). Only the B2-CH40-30-ST131 clone was associated with G3, accounting for 25.5% of G3 isolates, 10.7% of G2 isolates (*P* = 0.1068), and 0% of G1 isolates (*P* < 0.0001) (Supplementary Table S3).

### Comparison of the Virulence Factor (VF)-Encoding Genes Among G1, G2, and G3 Isolates

As indicated in Table 2, some VF-encoding genes were mostly observed among G1. These genes consisted of genes encoding adhesins (*papAH, papC, papEF, sfa/focDE*, and *yfcV*), genes encoding toxins (*cnf1, hlyA*, and *vat*), genes encoding two variants of group II capsule (*kpsM II-K5* and *neuC-K1*), and miscellaneous genes (*ibeA, malX*, and *ups*). Inversely, the *sat* and *traT* genes were mostly observed among G3 isolates. Concerning the genes encoding proteins involved in iron uptake, those encoding ferric aerobactin receptors (*iucD* and *iutA*) were significantly more frequent among G3 isolates than among G1 isolates, whereas it was the contrary for the *iroN* and *chuA* genes. Mean of VF-encoding genes was significantly higher among G1 isolates than among G3 isolates (13.8 vs. 10.4, *P* < 0.001).

**Table 2:** Virulence factor-encoding genes displayed by G1, G2, and G3 isolates.

Gene/Status	Number (%) of isolates				*P*-value	
	Total *n* = 394	G1 *n* = 36	G2 *n* = 28	G3 *n* = 330	G1 vs. G2	G1 vs. G3
**Adhesin**						
*fimH*	383 (97.2)	36 (100)	27 (96.4)	320 (97)		
*fimAVMT78*	56 (14.2)	4 (11.1)	4 (14.3)	48 (14.5)		
*papAH*	135 (34.3)	19 (52.8)	12 (42.9)	104 (31.5)		0.0149^b^
*papC*	140 (35.5)	20 (55.6)	12 (42.9)	108 (32.7)		0.0093^b^
*papEF*	146 (37.1)	20 (55.6)	13 (46.4)	113 (34.2)		0.0168^b^
*sfa/focDE*	69 (17.5)	22 (61.1)	9 (32.1)	38 (11.5)	0.0259^a^	<0.0001^b^
*afa/dra BC*	31 (7.9)	0 (0.0)	1 (3.6)	30 (9.1)		
*yfcV*	213 (54.1)	31 (86.1)	17 (60.7)	165 (50)	0.0396^a^	<0.0001^b^
**Toxin**						
*sat*	130 (33)	3 (8.3)	7 (25)	120 (36.4)		0.0006^b^
*cnf1*	74 (18.8)	17 (47.2)	12 (42.9)	45 (13.6)		<0.0001^b^
*hlyA*	80 (20.3)	17 (47.2)	13 (46.4)	50 (15.2)		<0.0001^b^
*hlyF*	77 (19.5)	6 (16.7)	4 (14.3)	67 (20.3)		
*cdtB*	17 (4.3)	4 (11.1)	0 (0.0)	13 (3.9)		
*tsh*	21 (5.3)	2 (5.6)	2 (7.1)	17 (5.2)		
*vat*	99 (25.1)	23 (63.9)	15 (53.6)	61 (18.5)		<0.0001^b^
**Iron uptake**						
*iucD*	237 (60.2)	8 (22.2)	13 (46.4)	216 (65.5)		<0.0001^b^
*IutA*	239 (60.7)	8 (22.2)	13 (46.4)	218 (66.1)		<0.0001^b^
*iroN*	129 (32.7)	25 (69.4)	14 (50)	90 (27.3)		<0.0001^b^
*fyuA*	295 (74.9)	29 (80.6)	21 (75)	245 (74.2)		
*chuA*	260 (66)	33 (91.7)	21 (75)	206 (62.4)		0.0008^b^
**Capsule**						
*KpsM II*	229 (58.1)	30 (83.3)	18 (64.3)	181 (54.8)		0.0011^b^
*KpsM II-K2*	31 (7.9)	0 (0.0)	1 (3.6)	30 (9.1)		
*KpsM II-K5*	151 (38.3)	21 (58.3)	13 (46.4)	117 (35.5)		0.0105^b^
*neuC-K1*	47 (11.9)	9 (25)	4 (14.3)	34 (10.3)		0.0153^b^
*KpsM III*	8 (2.0)	0 (0.0)	1 (3.6)	7 (2.1)		
**Miscellaneous**						
*cvaC*	58 (14.7)	6 (16.7)	3 (10.7)	49 (14.8)		
*iss*	72 (18.3)	8 (22.2)	3 (10.7)	61 (18.5)		
*traT*	256 (65)	15 (41.7)	13 (46.4)	228 (69.1)		0.0014^b^
*ibeA*	36 (9.1)	12 (33.3)	1 (3.6)	23 (7.0)	0.004^b^	<0.0001^b^
*malX*	226 (57.4)	31 (86.1)	17 (60.7)	178 (53.9)	0.0396^a^	0.0003^b^
*usp*	211 (53.6)	28 (77.8)	17 (60.7)	166 (50.3)		0.0024^b^
*ompT*	282 (71.6)	30 (83.3)	22 (78.6)	230 (69.7)		
**Range**	1–23	1–23	1–20	1–22		
**Mean**	10.8		11.8	10.4	0.001^c^	0.001^c^

*^*a*^Two-tailed *P*-values by Fisher’s exact test are shown where *P* < 0.05. ^*b*^Significant differences after applying the Bonferroni correction because of *P* < 0.025. ^*c*^Analysis of variance (ANOVA test) was performed to compare the mean of virulence genes between the three groups.*

### *fimB* Alleles

Given that the ST131 clone with a *fimH*30 allele was the dominant clone among our *E. coli* population and that previous results had shown (i) an *IS*3-like-linked defective *fimB* gene encoding a co-factor of the regulation of the type 1 fimbriae synthesis in this clone (Totsika et al., 2011) and (ii) the key role played by type 1 fimbriae in promoting biofilm formation in this clone (Sarkar et al., 2016), we analyzed the *fimB* gene in 127 isolates randomly selected among the 394 isolates. PCR experiments showed no *fimB* amplicon in 10 (7.9%) isolates (Supplementary Table S5) belonging to the following clones: A-CH11-0-ST93 (*n* = 2), A-CH11-0-ST167 (*n* = 1), A-CH99-54-ST361 (*n* = 1), A-CH7-53-ST540 (*n* = 1), A-CH7-54-ST540 (*n* = 1), A-CH4-0-ST1284 (*n* = 1), B1-CH4-0-ST155 (*n* = 1), B2-CH38-92-ST421 (*n* = 1), and E-CH26-0-ST38 (*n* = 1). An expected amplicon was detected in 89 (70.1%) isolates and an amplicon with a higher size was detected in 28 (22.0%) isolates (Supplementary Table S5). Sequencing experiments showed the insertion of *IS*3-like in the *fimB* gene in these 28 (22%) isolates comprising 25 of the 26 selected CH40-30 ST131 isolates and the three selected CH24-30 ST73 isolates. Overall, an intact *fimB* gene was significantly more frequent among G1 isolates than among G3 isolates (*P* = 0.03) (Supplementary Table S5).

**FIGURE 5:**
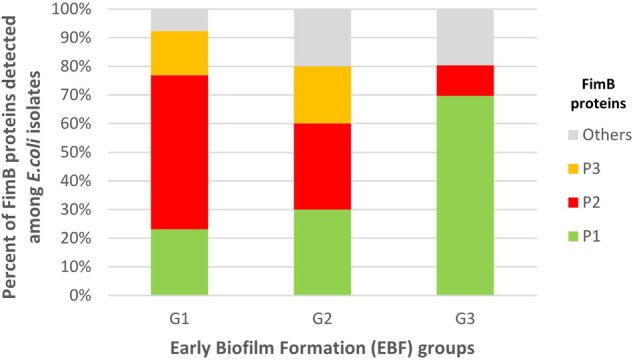
Distribution of the FimB protein (P) variants among G1, G2, and G3 isolates. G1: strong producers, G2: moderate producers, and G3: weak producers. FimB P2 and the closely related FimB P3 were associated with G1 and accounted for 69.2% of G1 isolates vs. 10.6% of G3 isolates (*P* < 0.0001). Inversely, FimB P1 was associated with G3 accounting for 69.7% of G3 isolates vs. 23.1% of G1 isolates (*P* = 0.0023).

Through *fimB* gene sequencing of the 89 isolates with an expected *fimB* amplicon, 34 *fimB* alleles were detected (Supplementary Table S6), of which 11 new variants compared with those currently registered in the GenBank database. These 34 alleles encoded 10 FimB proteins (P1 to P10) (Supplementary Table S6). Phylogenetic tree built with these 10 proteins (Supplementary Figure S2) revealed two large clusters. One of these clusters included the 14 alleles encoding FimB P1 and the other one included the eight alleles encoding FimB P2. FimB P2 and the closely related FimB P3 (Supplementary Figure S2) were associated with G1 and accounted for 69.2% of G1 isolates vs. 10.6% of G3 isolates (*P* < 0.0001). Inversely, FimB P1 was associated with G3, accounting for 69.7% of G3 isolates vs. 23.1% of G1 isolates (*P* = 0.0023) (Figure 5 and Supplementary Table S7).

### Correlation Between Biofilm Production and Bacterial Characteristics

The one-way ANOVA test using the individual BFI of 394 isolates showed that clonal lineage explained 72% of the EBF variability and FimB, 28%. Among the VF-encoding genes significantly associated with biofilm production, the *sfa/focDE, cnf1, hlyA, vat*, and *iroN* genes were those explaining from 8 to 16% of the variability of early biofilm production. Isolate source was not associated with the EBF.

## Discussion

Our study that assessed the early biofilm production, i.e., adhesion stage of 394 *E. coli* clinical isolates obtained from different sources, at the same time periods, in two distant hospitals (Spanish and French hospitals), is in accordance with previous studies with regard to the wide spectrum of biofilm formation within ExPEC (Ponnusamy et al., 2012; Agarwal et al., 2013; Mostafavi et al., 2018). Using the BioFilm Ring Test^®^ like us, Olivares et al. (2016) found that *P. aeruginosa* causing infection in cystic fibrosis patients segregated into three groups (strong, moderate, and weak producers) at the early stage of biofilm formation. Accordingly, difference in ability to quickly adhere seems to exist within isolates of different bacterial species. The biofilm production displayed by our ESBLEC collection is also in accordance with that displayed by the ESBLEC collection recently published by Surgers et al. (2018), i.e., predominance of weak biofilm producers among ESBLEC.

The novelty provided by our study consisted of the molecular characterization of the 394 isolates with regard to phylogroups, clonal lineages, VF-encoding genes, and FimB, a co-factor of the regulation of the synthesis of type 1 fimbriae that are critical adhesins for *E. coli* biofilm formation (Beloin et al., 2008). Thus, we showed that among the characteristics significantly associated with EBF, clonal lineage was the most suitable characteristics to explain the variability of EBF. Association between clonal lineage and EBF phenotype has recently been shown among bone and joint infection *Staphylococcus aureus* isolates analyzed by using the BioFilm Ring Test^®^ (Tasse et al., 2018).

Among the limited number (<10%) of our *E. coli* that quickly and persistently produced early biofilm at high levels (G1 isolates), five clones (B2-CH14-2-ST127, CH52-5, and CH52-14 sub-lineages of B2-ST141, B2-CH40-22-ST131, and E-CH100-96-ST362) accounted for 41.7% of the 36 G1 isolates. Notable features have previously been provided by different studies for these clones. The B2-ST127 clone, which is one of the dominant clones among uropathogenic isolates (Yamaji et al., 2018), was shown to be shared by humans, dogs, and cats (Johnson et al., 2008) and to be a dominant gut colonizer of humans (Ulleryd et al., 2015) and fruit bats (Nowak et al., 2017). The B2-ST141 clone was characterized by Clermont et al. (2017) as a commensal clone, i.e., digestive tract resident with low level of human invasiveness, through a study assessing the pathogenesis of bacteriaemic *E. coli* by matching large collections of bacteriaemic (Lefort et al., 2011) and commensal isolates (Massot et al., 2016). Other studies showed that this clone accounted for the gut dominant *E. coli* population of some healthy subjects (Leflon-Guibout et al., 2008; Nicolas-Chanoine et al., 2013) and was one of the dominant clones in the digestive tract of Antarctic pinnipeds (Mora et al., 2018). The B2-CH40-22 ST131 clone was shown to have similar features to the B2-CH52-5 ST141 clone with regard to healthy subjects (Nicolas-Chanoine et al., 2017) and Antarctic pinnipeds (Mora et al., 2018). It was also found in poultry digestive tract and retail chicken meat and was shown to be a foodborne uropathogen (Liu et al., 2018). Concerning the E-CH100-96 ST362 clone, our study is, to our knowledge, the first one reporting the presence of this clone in humans. Indeed, it was identified so far in chicken (Cortes et al., 2010) and as an agent causing bovine mastitis (Freitag et al., 2017). Accordingly, most of these clones appear to be both ExPEC and intestinal dominant colonizers in some mammals and birds.

We found that some VF genes, including *papC, sfa/focDE, cnf1, hlyA*, and *ibeA*, were mostly identified in G1 isolates, i.e., strong biofilm producers. All these VFs had already been shown to be associated with *E. coli* strong biofilm producers (Naves et al., 2008), and two of them, *sfa/focDE* and *ibeA*, had been shown to be characteristic traits of adherent/invasive *E. coli* (AIEC) that are strong biofilm producers (Martinez-Medina et al., 2009b). The implication of AIEC in Crohn’s disease (Darfeuille-Michaud et al., 2004) was notably based on their high biofilm production. Indeed, in an inflamed intestinal environment, their biofilm-related mucosal attachment could allow them to penetrate the inner mucus layer and adhere to the epithelial surface, thus enabling more direct stimulation of epithelial and immune cells (Glasser et al., 2001; Ellermann and Sartot, 2018). It is interesting to note here that Martinez-Medina et al. (2009a) found two ST131 isolates carrying the *ibeA* gene in the AIEC collection that they compared with ExPEC isolates. This finding suggests that it would be of interest to investigate all the AIEC phenotypic and genotypic features in our strong biofilm producers (Camprubí-Font et al., 2019).

The present study in which a wide collection of *E. coli* isolates was analyzed confirms the results that we previously obtained with few isolates about the difference in early biofilm production between B2-CH40-22 ST131 and B2-CH40-30-ST131 clones, i.e., strong and weak production, respectively (Nicolas-Chanoine et al., 2017). Knowing that the B2-CH40-22 ST131 clone is the ancestor of the globally disseminated B2-CH40-30-ST131 clone (Ben Zakour et al., 2016), the loss of the ability to strongly produce biofilm at the early step of biofilm formation by the B2-CH40-30-ST131 clone could appear as an evolutionary adaptative trait in ST131 lineage.

In conclusion, this study highlights that the ability to quickly and persistently produce biofilm at high levels is a property displayed by a limited number of clones, some of which have been found in previous studies as dominant colonizers in some mammals and birds.

## Ethics Statement

Ethics approval was not required according to national and institutional guidelines.

## Author Contributions

S-CF-S carried out biofilm assay and strain molecular characterizations, collected and analyzed the all data, and participated in manuscript writing. MD and NM collected and phenotypically characterized the French clinical isolates, and participated in both biofilm assay and strain molecular characterizations. VG participated in strain molecular characterizations. MA participated in isolation and characterization of the Spanish clinical isolates. JB supervised the strain molecular analyses, data collection, and statistical analyses, and participated in manuscript writing. M-HN-C designed the research, supervised the overall project, and wrote the manuscript. All authors provided critical input and approved the manuscript submission.

## Conflict of Interest Statement

The authors declare that the research was conducted in the absence of any commercial or financial relationships that could be construed as a potential conflict of interest.

## Footnotes

1 http://mlst.warwick.ac.uk/mlst/dbs/Ecoli

2 https://www.xlstat.com
