# Supplementary material for: Association Between Kinetics of Early Biofilm Formation and Clonal Lineage in Escherichia coli
Source: Front Microbiol. 2019 May 31;10:1183. doi: 10.3389/fmicb.2019.01183 (PMC6555128; doi:10.3389/fmicb.2019.01183)
Supplement: Supplementary file 1 [file Data_Sheet_1.PDF]

Association between kinetic of early biofilm formation and clonal lineage in *Escherichia coli*

Saskia-Camille Flament Simon[1], Marion Duprilot[2, 3] Noémie Mayer[2], Vanesa García [1], María Pilar Alonso[4], Jorge Blanco[1], Marie-Hélène Nicolas-Chanoine[3]

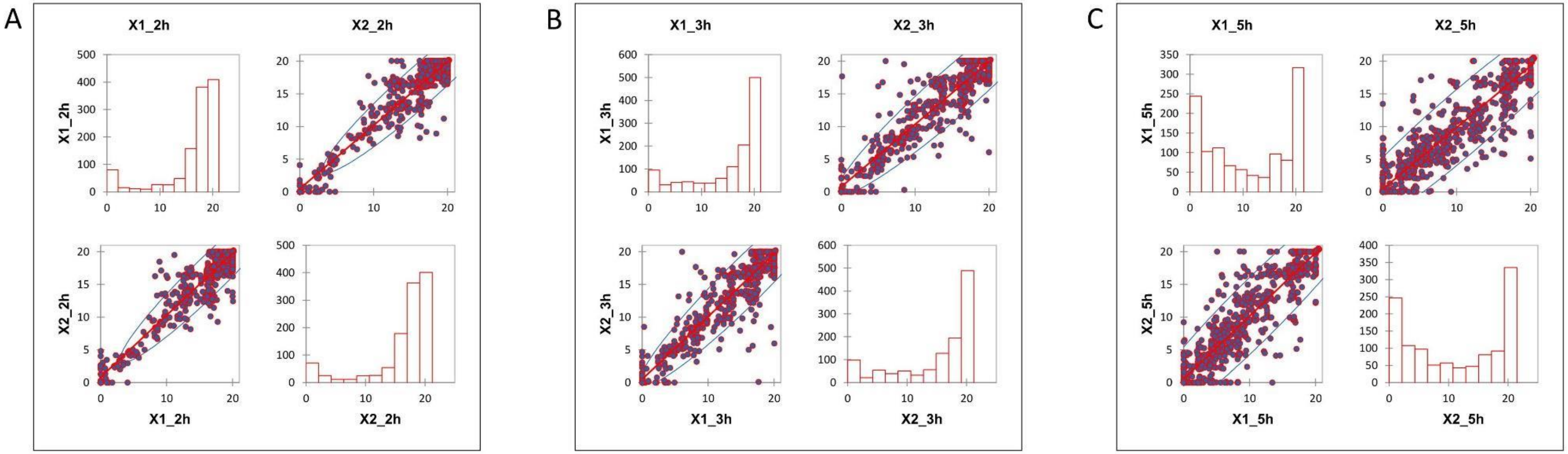

Figure S1A. **Pearson´s correlation between the BFI values obtained for each of the 394 *E. coli* isolates.** A: 0.96 at 2h, B: 0.96 at 3h and C: 0.95 at 5h.

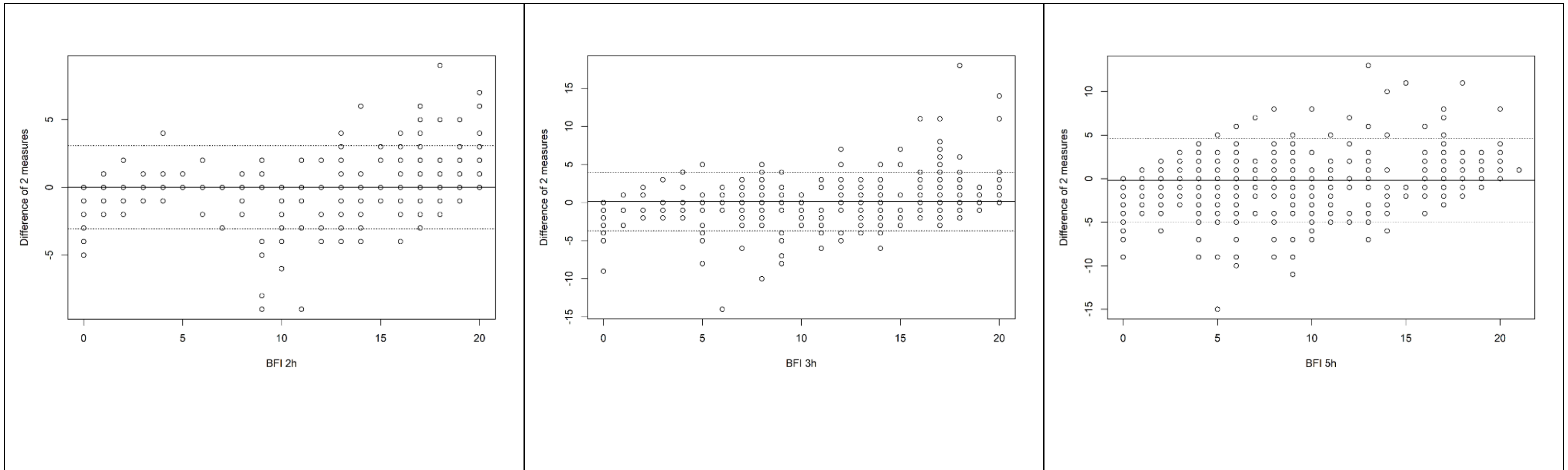

Figure S1B. Assessment of repeatability between the BFI values obtained for each of the 394 *E. coli* isolates using the Bland and Altman statistical method (Bland and Altman, 1986). The mean of individual BFI measures (continuous line) and standard deviation (dashed line) representing ±2SD from mean line were calculated at 2, 3 and 5h. Only 3.4% of values at 2 h, 5.5% at 3 h and 6.8% at 5h exceed the established standard deviation.

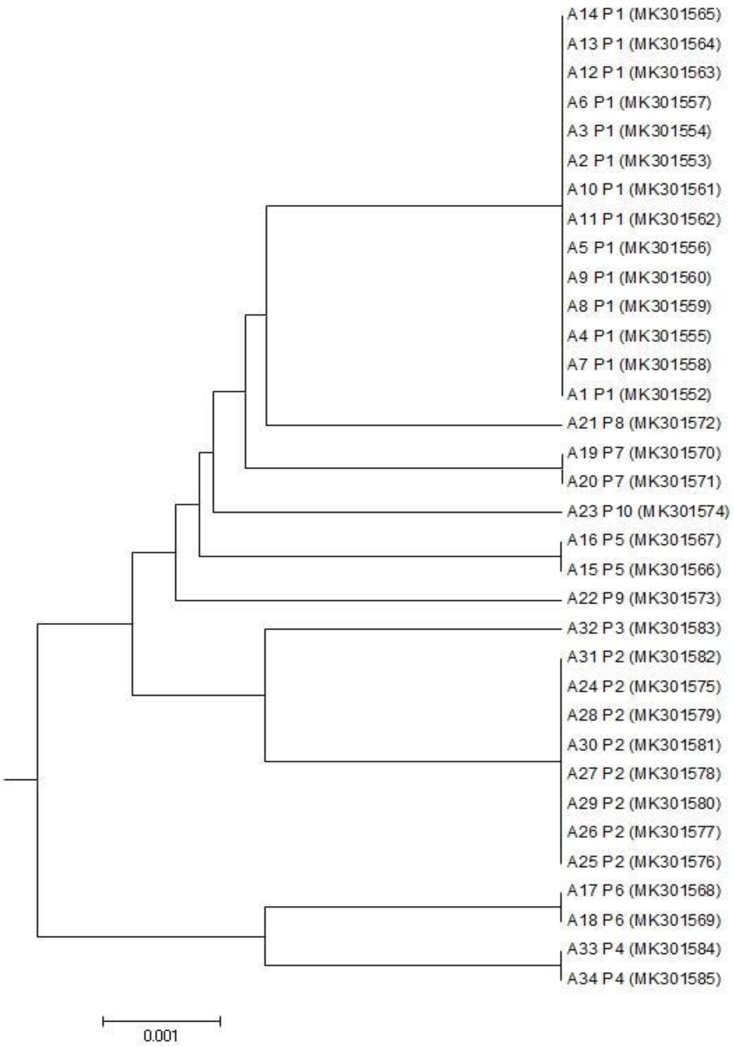

Figure S2. Phylogenetic tree of FimB (sequences available in Table S1).

In parenthesis are indicated the access numbers of the sequences registered in GenBank database.

Table S1. Distribution of phylogroups A, B1, B2, C, D, E and F among G1, G2 and G3 isolates.

Phylogroup	Number (%) of isolates				*P* value[a]
	Total (n=394)	G1 (n=36)	G2 (n=28)	G3 (n=330)	G1 *vs* G3
A	59 (15.0)	0 (0)	6 (21.4)	53 (16.1)	0.0098
B1	38 (9.6)	3 (8.3)	1 (3.6)	34 (10.3)	
B2	202 (51.3)	28 (77.8)	17 (60.7)	157 (47.6)	0.0006
C	38 (9.6)	0 (0)	0 (0)	38 (11.5)	0.0419
D	17 (4.3)	0 (0)	4 (11.1)	13 (3.9)	
E	20 (5.1)	2 (5.6)	0 (0)	18 (5.4)	
F	20 (5.1)	3 (8.3)	0 (0)	17 (5.2)	

[a] Two-tailed *P* values by Fisher´s exact test are shown where $P<0.05$.

Table S2. Distribution of the most frequent sequence types (STs) among G1, G2 and G3 isolates.

Sequence Type	Number (%) of isolates				*P* value[a]
	Total (n=394)	G1 (n=36)	G2 (n=28)	G3 (n=330)	G1 *vs* G3
ST10	17 (4.3)	0 (0)	2 (7.1)	15 (4.5)	
ST69	15 (3.8)	0 (0)	3 (10.7)	12 (3.6)	
ST73	22 (5.6)	4 (11.1)	5 (17.9)	13 (3.9)	
ST88	17 (4.3)	0 (0)	0 (0)	17 (5.2)	
ST95	14 (3.6)	0 (0)	0 (0)	14 (4.2)	
ST131	104 (26.4)	4 (11.1)	3 (10.7)	97 (29.4)	0.0289
ST141	16 (4.1)	9 (25)	2 (7.1)	5 (1.5)	<0.0001
Remaining-ST	189 (48.0)	19 (57.8)	13 (46.4)	157 (47.6)	

[a] Two-tailed *P*values by Fisher´s exact test are shown where *P* <0.05

Table S3. Distribution (%) of the 31 clones including at least three isolates into G1, G2 and G3.

Clone	Number (%) of isolates			*P* value[a]
	G1 (n=36)	G2 (n=28)	G3 (n=330)	G1*vs* G3
A-CH11-54-ST10	0	1 (3.6)	9 (2.7)	
A-CH11-0-ST93	0	0	4 (1.2)	
A-CH11-0-ST167	0	1 (3.6)	2 (0.6)	
A-CH99-54-ST361	0	0	5 (1.5)	
A-CH11-54-ST744	0	0	5 (1.5)	
B1-CH4-27-ST58	0	0	4 (1.2)	
B1-CH6-31-ST453	0	0	3 (0.9)	
B2-CH13-106-ST12	1 (2.8)	1 (3.6)	2 (0.6)	
B2-CH24-10-ST73	1 (2.8)	2 (7.1)	3 (0.9)	
B2-CH24-103-ST73	0	1 (3.6)	5 (1.5)	
B2-CH24-12-ST73	0	1 (3.6)	2 (0.6)	
B2-CH24-30-ST73	1 (2.8)	1 (3.6)	2 (0.6)	
B2-CH38-15-ST95	0	0	6 (1.8)	
B2-CH14-2-ST127	2 (5.6)	1 (3.6)	1 (0.3)	0.0265
B2-CH40-22-ST131	3 (8.3)	0	1 (0.3)	0.0033
B2-CH40-30-ST131	0	3 (10.7)	84 (25.5)	<0.0001
B2-CH40-41-ST131	0	0	10 (3)	
B2-CH52-5-ST141	6 (16.7)	2 (7.1)	5 (1.5)	0.0002
B2-CH14-27-ST404	0	0	3 (0.9)	
B2-CH14-64-ST1193	0	0	4 (1.2)	
C-CH4-35-ST23	0	0	3 (0.9)	
C-CH4-39-ST88	0	0	11 (3.3)	
C-CH4-24-ST410	0	0	7 (2.1)	
C-CH263-32-ST1615	0	0	6 (1.8)	
D-CH35-27-ST69	0	3 (10.7)	11 (3.3)	
E-CH26-0-ST38	0	0	3 (0.9)	
E-CH100-96-ST362	2 (5.6)	0	1 (0.3)	0.0265
E-CH37-27-ST405	0	0	4 (1.2)	
F-CH32-41-ST59	0	0	4 (1.2)	
F-CH45-97-ST117	0	0	3 (0.9)	
F-CH88-58-ST354	0	0	5 (1.5)	

[a] Two-tailed *P*values by Fisher´s exact test are shown where *P* <0.05.

Table S4. Distribution of the 26 G1-detected clones among G1, G2 and G3 isolates

Clone	Number (%) of isolates			*P* value[a]	
	G1 (n=36)	G2 (n=28)	G3 (n=330)	G1 *vs*G2	G1 *vs*G3
B1-CH23-54-ST205	1 (2.8)	0	0		
B1-CH6-31-ST1196	1 (2.8)	0	0		
B1-CH4-31-ST2766	1 (2.8)	0	0		
B2-CH13-106-ST12	1 (2.8)	1 (3.6)	2 (0.6)		
B2-CH13-223-ST12	1 (2.8)	0	0		
B2-CH24-10-ST73	1 (2.8)	2 (7.1)	3		
B2-CH24-13-ST73	1 (2.8)	0	0		
B2-CH24-30-ST73	1 (2.8)	1 (3.6)	2 (0.6)		
B2-CH24-32-ST73	1 (2.8)	0	0		
B2-CH24-2-ST104	1 (2.8)	0	0		
B2-CH14-2-ST127	2 (5.6)	1 (3.6)	1 (0.3)		0.0265
B2-CH40-22-ST131	3 (8.3)	0	1 (0.3)		0.0033
B2-CH40-298-ST131	1 (2.8)	0	0		
B2-CH40-22-ST2556	1 (2.8)	0	0		
B2-CH52-5-ST141	6 (16.7)	2 (7.1)	5 (1.5)		0.0002
B2-CH52-14-ST141	2 (5.6)	0	0		0.0094
B2-CH52-76-ST141	1 (2.8)	0	0		
B2-CH38-5-ST569	1 (2.8)	0	0		
B2-CH108-75-ST636	1 (2.8)	0	0		
B2-CH24-12-ST1154	1 (2.8)	0	0		
B2-CH319-197-ST2015	1 (2.8)	0	0		
B2-CH43-0-ST2558	1 (2.8)	0	0		
E-CH100-96-ST362	2 (5.6)	0	1 (0.3)		0.0265
F-CH4-171-ST648	1 (2.8)	0	1 (0.3)		
F-CH4-58-ST648	1 (2.8)	0	1 (0.3)		
F-CH231-58-ST1485	1 (2.8)	0	0		
	36 (100)	7 (25)	17 (5.2)	<0.0001	<0.0001

[a] Two-tailed *P*values by Fisher´s exact test are shown where *P*<0.05

Table S5 *fimB* gene status in G1, G2 and G3 isolates.

fimB gene status	Number (%) of isolates				*P* value[a]
	Total (n=127)	G1 (n=14)	G2 (n=12)	G3 (n=101)	G1 *vs* G3
Intact	89 (70.1)	13 (92.9)	10 (83.3)	66 (65.3)	0.0301
Disrupted	28 (22.0)	1 (7.1)	1 (8.3)	26 (25.7)	
Absent	10 (7.9)	0	1 (8.3)	9 (8.9)	

[a] One-tailed *P*values by Fisher´s exact test are shown where $P<0.05$.

Table S6. Nucleotide and amino acid differences in 34 *fimB* alleles detected in 84 *Escherichia coli* clinical isolates.

Strain/FimB/accession n°	Nucleotide position																																							Amino acid position							
	39	57	72	80	93	103	114	117	156	198	237	246	249	256	285	294	297	315	319	324	330	339	345	384	396	402	485	495	498	519	537	540	556	570	573	579	580	#		27	35	86	107	162	180	186	195
A1 P1 (MK301552)	G	A	A	C	A	T	T	G	T	A	C	G	T	G	C	G	C	G	G	T	A	G	G	C	C	G	A	T	G	T	C	T	G	A	C	A	C	C		T	Y	V	V	Q	N	G	Q
A22 P9 (MK301573)	.	.	.	.	.	.	.	.	.	.	.	.	.	.	.	.	.	.	A	.	.	.	.	.	.	.	.	.	.	.	.	.	.	.	.	.	.	.		.	.	.	I	.	.	.	.
A7 P1 (MK301558)	.	.	.	.	.	.	.	.	.	.	.	.	C	.	.	.	.	A	.	.	G	A	.	.	.	.	.	.	.	.	.	.	.	.	.	.	.	.		.	.	.	.	.	.	.	.
A16 P5 (MK301567)	.	.	.	.	.	.	.	.	.	.	.	.	C	.	.	.	.	A	.	.	G	A	.	.	.	.	.	.	.	.	.	A	.	.	.	.	.	.		.	.	.	.	.	K	.	.
A4 P1 (MK301555)	.	.	.	.	.	.	.	.	.	.	.	.	C	.	.	.	.	A	.	.	G	.	.	.	.	.	.	.	.	.	.	.	.	.	.	.	.	.		.	.	.	.	.	.	.	.
A8 P1 (MK301559)	.	.	.	.	.	.	.	.	.	.	.	.	C	.	.	.	.	A	.	.	.	.	.	.	.	.	.	.	.	.	.	.	.	.	.	.	.	.		.	.	.	.	.	.	.	.
A9 P1 (MK301560)	.	.	.	.	.	.	.	.	.	.	.	.	C	.	.	.	.	.	.	.	G	.	.	.	.	.	.	G	.	.	.	.	.	.	.	.	.	.		.	.	.	.	.	.	.	.
A23 P10 (MK301574)	.	.	.	A	.	.	.	.	.	.	.	.	C	.	.	.	.	.	.	.	G	.	.	.	.	.	.	.	.	.	.	.	.	.	.	.	.	.		N	.	.	.	.	.	.	.
A5 P1 (MK301556)	.	.	.	.	.	.	.	.	.	.	.	.	C	.	.	.	.	.	.	.	G	.	.	.	.	.	.	.	.	.	.	.	.	.	.	.	.	.		.	.	.	.	.	.	.	.
A15 P5 (MK301566)	.	.	.	.	.	.	.	.	.	.	T	.	C	.	.	.	.	.	.	.	T	A	.	.	.	.	.	.	.	.	.	A	.	.	.	.	.	.		.	.	.	.	.	K	.	.
A17 P6 (MK301568)	.	.	.	.	.	C	.	.	.	.	T	.	C	.	.	.	.	.	.	.	T	A	.	.	.	.	.	.	.	.	.	.	.	.	.	.	.	.		.	H	.	.	.	.	.	.
A11 P1 (MK301562)	.	.	.	.	.	.	.	.	.	.	.	.	C	.	.	.	.	.	.	.	T	A	.	.	.	.	.	.	.	.	.	.	.	.	.	.	.	.		.	.	.	.	.	.	.	.
A10 P1 (MK301561)	.	.	.	.	.	.	.	.	.	.	T	.	C	.	.	.	.	.	.	.	T	.	.	.	.	.	.	.	.	.	.	.	.	.	.	.	.	.		.	.	.	.	.	.	.	.
A2 P1 (MK301553)	.	.	.	.	.	.	.	.	.	.	T	.	C	.	.	.	.	.	.	.	T	A	.	.	.	.	.	.	.	.	.	.	.	.	.	.	.	.		.	.	.	.	.	.	.	.
A19 P7 (MK301570)	.	.	.	.	.	.	.	.	.	C	.	.	C	.	.	.	.	A	.	.	G	.	.	.	.	.	.	.	.	.	.	.	A	G	.	.	.	.		.	.	.	.	.	.	S	.
A3 P1 (MK301554)	.	.	.	.	.	.	.	.	.	C	.	.	C	.	.	.	.	A	.	.	G	.	.	.	.	.	.	.	.	.	.	.	.	G	.	.	.	.		.	.	.	.	.	.	.	.
A18 P6 (MK301569)	.	.	.	.	.	C	.	.	.	.	.	.	C	.	.	T	T	.	.	.	G	.	.	.	.	.	.	.	.	.	.	.	.	.	.	.	.	.		.	H	.	.	.	.	.	.
A6 P1 (MK301557)	.	.	.	.	.	.	.	.	.	.	.	.	C	.	.	T	T	.	.	.	.	.	.	T	.	.	.	.	.	.	.	.	.	.	.	G	A	.		.	.	.	.	.	.	.	.
A12 P1 (MK301563)	.	.	.	.	.	.	.	.	.	.	.	.	C	.	.	T	T	.	.	.	.	.	.	T	.	.	.	.	.	.	.	.	.	.	.	.	.	.		.	.	.	.	.	.	.	.
A20 P7 (MK301571)	.	.	.	.	.	.	.	.	.	.	.	.	C	.	.	T	T	.	.	.	.	.	.	T	.	.	.	.	.	.	.	.	A	.	.	.	.	.		.	.	.	.	.	.	S	.
A21 P8 (MK301572)	.	.	.	.	.	.	.	.	.	.	.	A	C	.	.	T	T	.	.	.	.	.	.	T	.	.	G	.	.	.	.	.	.	.	.	.	.	.		.	.	.	.	R	.	.	.
A31 P2 (MK301582)	T	.	.	.	T	.	.	.	C	.	.	.	C	A	.	T	T	.	.	.	.	.	A	.	.	.	.	.	.	.	.	.	.	.	.	.	.	.		.	.	I	.	.	.	.	.
A24 P2 (MK301575)	.	.	T	.	T	.	A	A	C	.	.	.	C	A	.	T	T	.	.	.	.	.	A	.	.	.	.	.	A	.	.	.	.	.	.	.	.	.		.	.	I	.	.	.	.	.
A33 P4 (MK301584)	.	.	T	.	T	C	A	A	C	.	.	.	C	A	.	T	T	.	.	.	.	.	A	.	.	.	.	.	A	.	.	.	.	.	.	.	.	.		.	H	I	.	.	.	.	.
A34 P4 (MK301585)	.	.	.	.	T	C	A	A	C	.	.	.	C	A	.	T	T	.	.	.	.	.	A	.	.	.	.	.	A	.	.	.	.	.	.	.	.	.		.	H	I	.	.	.	.	.
A32 P3 (MK301583)	.	.	.	.	T	.	A	A	C	.	.	.	C	A	.	T	T	.	.	.	.	.	A	.	.	.	.	.	A	.	.	.	.	.	.	.	.	G		.	.	I	.	.	.	.	E
A28 P2 (MK301579)	.	.	.	.	T	.	A	A	C	.	.	.	C	A	.	T	T	.	.	C	.	.	A	.	.	.	.	.	A	.	.	.	.	.	.	.	.	.		.	.	I	.	.	.	.	.
A30 P2 (MK301581)	.	.	.	.	T	.	A	A	C	.	.	.	C	A	.	T	T	.	.	C	.	.	A	.	.	.	.	.	.	.	.	.	.	.	.	.	.	.		.	.	I	.	.	.	.	.
A27 P2 (MK301578)	.	.	.	.	T	.	A	A	C	.	.	.	C	A	.	T	T	.	.	.	.	.	.	.	.	.	.	.	A	.	.	.	.	.	.	.	.	.		.	.	I	.	.	.	.	.
A29 P2 (MK301580)	.	.	.	.	T	.	A	A	C	.	.	.	C	A	.	T	T	.	.	.	.	.	.	.	.	.	.	.	.	.	.	.	.	.	.	.	.	.		.	.	I	.	.	.	.	.
A26 P2 (MK301577)	.	.	.	.	T	.	A	A	C	.	.	.	C	A	.	T	T	.	.	.	.	.	A	.	.	.	.	.	A	.	.	.	.	.	.	.	.	.		.	.	I	.	.	.	.	.
A25 P2 (MK301576)	.	.	.	.	T	.	A	A	C	.	.	.	C	A	.	T	T	.	.	.	.	.	A	.	.	.	.	.	.	.	.	.	.	.	.	.	.	.		.	.	I	.	.	.	.	.
A14 P1 (MK301565)	T	G	.	.	.	.	.	.	.	.	.	.	C	.	A	T	T	.	.	.	.	.	.	T	T	A	.	.	.	.	T	.	.	.	.	G	A	.		.	.	.	.	.	.	.	.
A13 P1 (MK301564)	.	G	.	.	.	.	.	.	.	.	.	.	C	.	.	.	T	.	.	.	.	.	.	T	T	A	.	.	.	A	.	.	.	.	T	G	A	.		.	.	.	.	.	.	.	.

Table S7. Distribution of FimB variants in G1, G2 and G3 isolates.

FimB variant	Number (%) of isolates				*P* value[a]
	Total (n=89)	G1 (n=13)	G2 (n=10)	G3 (n=66)	G1 *vs* G3
P1	52 (58.4)	3 (23.1)	3 (30)	46 (69.7)	0.0023
P2	17 (19.1)	7 (53.8)	3 (30)	7 (10.6)	0.0011
P3	4 (4.5)	2 (15.4)	2 (20)	0	0.0253
P2 or P3	21 (23.6)	9 (69.2	5 (50)	7 (10.6)	<0.0001
Others	16 (18.0)	1 (7.7)	2 (20)	13 (19.7)	

[a] Two-tailed *P* values by Fisher´s exact test are shown where *P*<0.05.
